# Inter- and Intra-Specific Density-Dependent Effects on Life History and Development Strategies of Larval Mosquitoes

**DOI:** 10.1371/journal.pone.0057875

**Published:** 2013-03-04

**Authors:** Ido Tsurim, Alon Silberbush, Ofer Ovadia, Leon Blaustein, Yoel Margalith

**Affiliations:** 1 Center for Biological Control, Department of Life Sciences, Ben-Gurion University of the Negev, Beer Sheva, Israel; 2 Department of Life Sciences, Achva Academic College, MP Shikmim, Israel; 3 Kadas Green Roofs Ecology Center and Community Ecology Laboratory, Institute of Evolution and Department of Evolutionary and Environmental Biology, Faculty of Sciences, University of Haifa, Haifa, Israel; Texas A&M University, United States of America

## Abstract

We explored how inter- and intra-specific competition among larvae of two temporary-pool mosquito species, *Culiseta longiareolata* and *Ochlerotatus caspius*, affect larval developmental strategy and life history traits. Given that their larvae have similar feeding habits, we expected negative reciprocal inter-specific interactions. In a microcosm experiment, we found sex-specific responses of larval survival and development to both intra- and inter-specific larval competition. *C. longiareolata* was the superior competitor, reducing adult size and modifying larval developmental time of *O. caspius*. We observed two distinct waves of adult emergence in *O. caspius*, with clear sex-specific responses to its inter-specific competitor. In males, this pattern was not affected by *C. longiareolata*, but in females, the timing and average body size of the second wave strongly varied with *C. longiareolata* density. Specifically, in the absence of *C. longiareolata*, the second wave immediately followed the first wave. However, as *C. longiareolata* abundance increased, the second wave was progressively delayed and the resulting females tended to be larger. This study improves our understanding of the way intra- and inter-specific competition combine to influence the life histories of species making up temporary pond communities. It also provides strong evidence that not all individuals of a cohort employ the same strategies in response to competition.

## Introduction

Competitive interactions can strongly influence community structure and function [1]–[4]. At the heart of such effects are impacts of intra- and inter-specific competition on behavior, reproduction, and life history traits [3]–[7]. In organisms with complex life cycles, larval resource allocation to life history traits and maintenance can be facultative. Hence, larval development strategy may be modified in response to environmental stressors, such as resource competition [3], [4], [8]–[13].

In mosquitoes, competition for food and space during larval stages can influence a multitude of fitness-related life history traits. Increased intra-specific larval densities have been shown to cause reduced larval survival, prolonged larval development, and reduced larval metabolic rate [14]–[20]. Increased larval density has been shown to cause decreased adult size and survival [16], [19], [21], delayed time to first blood meal, increased number of required blood meals for development of a full clutch of eggs, reduced autogenic capabilities, and reduced female fecundity [16], [18]. Females emerging from high-density larval conditions may also exhibit increased tendency to disperse [16]. Unlike intra-specific competition, less attention has been devoted to trait-mediated (life history) effects of inter-specific competition among mosquito species [4], [22]. Experimental work on inter-specific larval interactions has been largely limited to a small number of mosquito species that breed in container habitats (such as phytotelmata), usually focusing on species coexistence, community composition and stability, and species richness and diversity [4], [22].

In this study, we focus on *Ochlerotatus caspius* and *Culiseta longiareolata*, two of the most common mosquito species in temporary aquatic breeding sites in the Dead Sea basin [23]–[25]. Both species are early colonizers of flood-filled and rain-filled pools after inundation. The larvae of *O. caspius* are euryhaline and are found in a wide range of aquatic habitats [23], [26]. *C. longiareolata* larvae are restricted to freshwater pools [26]. Larvae of the two species often co-occur in temporary freshwater habitats [24], [26]–[28]. *O. caspius* is widely distributed throughout North Africa, the Middle East, Europe and Central Asia ([29], [30]). It is a potential vector for several human arboviruses ([31]–[34]), and an important nuisance pest throughout its distribution range ([35]). The female is considered a generalist, taking blood meals from a wide range of vertebrates. The eggs are oviposited singly or in small clusters on the water surface or above it in the surrounding wet soil ([27], [35], personal observations). The eggs may hatch within a few days, or if under dry conditions, may enter diapause until the next inundation. *C. longiareolata* is one of the common mosquito species in the Mediterranean and Negev regions of Israel ([24], [25], [36], [37]). The female takes its blood meals mostly from birds, and oviposits its entire clutch into a single egg raft on the water surface ([38]). Its rapid colonization ability by oviposition (often within hours of a flooding event and before most other species), the competitive superiority of its larvae, and their role as intra-guild predators, suggest *C. longiareolata* as an important species in the temporary pond ecosystem [39]–[43]. In this study, we examined whether competition between *C. longiareolata* and *O. caspius* occurs and if so, how it translates into fitness effects through larval development strategy and survival. Since larval *C. longiareolata* are strong competitors, we expected asymmetric competitive interactions between the two species, namely, a stronger effect of *C. longiareolata* on *O. caspius* than the reciprocal effect.

Larval mosquitoes, being confined to their oviposition site, have a restricted capacity for a spatial response to increased competition. All else being equal, the number and phenotypes of larval mosquitoes reaching adulthood in a certain pool should reflect the intra- and inter-specific competitive interactions throughout the larval development period. In two-species competitive systems, the equilibrium densities (or biomass) of the two competing species should be negatively correlated. If competition is asymmetric, the slope of this relationship, with the stronger competitor in the ordinate, is expected to be smaller than minus one (−1). We also expect that larval development strategy will change with respect to the competitive environment. Specifically, we expected extended larval developmental time and reduced adult size, proportional to the relevant competitive intensities, with *O. caspius* affected more by *C. longiareolata* than vice versa.

## Methods

### Ethics Statement

No specific permits were required for the described field collections and lab studies.

*C. longiareolata* larvae originated from nine egg rafts collected in the field (Southern Dead Sea Basin 30°56′ N 35°24′E), one day before the onset of the experiment. The egg rafts were transferred to the lab, at Ben-Gurion University, Beer Sheva, Israel, and hatched in aged tap water. *O. caspius* larvae originated from eggs extracted from a two generation old, laboratory colony, established from larvae collected at the same location as the *C. longiareolata* egg rafts. Eggs were hatched in lab in aged tap water one day prior to the experiment. We allowed temperature to vary with natural ambient temperature. All larvae experienced the same conditions from hatching and throughout the experiment as follows: photoperiod was 13∶11 light:dark hours, and temperatures in the water ranged from 18 to 30°C with an average daily minimum of 23°C (se = 0.3) and daily maximum of 28°C (se = 0.3).

Field collection of egg rafts was done on public property and did not require any permits or specific permissions. Both study species are not endangered and/or protected by law. No permits are required for their study and field collection.

We used 500 ml cups, filled with 400 ml of aged tap water (water depth: ∼6 cm; water surface area: ∼66.6 cm^2^) as laboratory microcosms for our experiment. Small water bodies of this size are within the range utilized by both *C. longiareolata* and *O. caspius* larvae (I. Tsurim, personal observations) and microcosms of this size are commonly used to study mosquito larval interactions (e.g., [44]). To each cup, we added 60 mg of a finely ground mixture of “Sera pond” bio-flakes and “Kopoleh” rodent chow (21.4% protein) three times during the experiment - at the onset of the experiment, at day 7 and at day 20.

### Experimental Design

We compared larval performance under five different proportions of *C. longiareolata* and *O. caspius* (treatment: initial larval combinations; Table 1). Larvae hatched from different egg rafts (*C. longiareolata*) or egg clutches (*O. caspius*) were combined and then randomly distributed among experimental treatments and replicates (cups). We used a replacement design (e.g. see [13], [45]), in which all cups received a total of 80 1^st^ instar larvae. Each combination (treatment) was replicated six times (total cups = 30; total larvae = 2400). Cups were randomly assigned to treatment and were rotated each morning to reduce any possible variance due to cup location. Evaporated water was replenished daily with aged tap water.

**Table 1:** Initial abundance combinations of 1^st^ instar larvae, each having six replicates.

Treatment	*Ochlerotatus caspius*	*Culiseta longiareolata*
A	80	0
B	0	80
C	40	40
D	20	60
E	60	20

#### Survival and larval development time

Pupae were removed each morning and placed in separate vials until emergence. The time from introduction of first instar larvae to adult emergence (larval development time) was noted. The adults were identified to species and sex and kept in freezer (−30°C) for subsequent measurements.

#### Adult size

Adult size measurements included dry weight, wing length (the distance from the axillary incision to the apical margin, excluding fringe scales; [46]), hind femur length, and hind tibia length. The mosquitoes were first dried at 40°C for 24 hr. Individual mosquitoes were then weighed to the nearest 10^−5 ^g using *Precisa 40SM-200A* balance. Next, we measured the wings and hind legs of individuals on a microscope slide containing an embedded scale, by photographing them using a digital camera (Micropublisher 5.0, QImaging, Surrey, BC, Canada) connected to a Nikon stereoscope (SMZ 800, Nikon, Kawaski, Japan), and then measuring to the nearest 10^−3^ mm, using ImageJ software [47]. In our analyses, we used the average wing, femur and tibia length measurements (average taken from both sides of each individual).

### Statistical Analysis

We applied standard general linear models to analyze relationships between treatment (initial larval combinations) and final adult abundances and biomass. We tested the assumptions of the standard tests concerning normal distribution of the data, using Kolmogorov-Smirnov Chi square goodness of fit, and homogeneity of the variance, using Levene’s test. Unless stated otherwise, the assumptions were met and the statistical analysis was applied on the raw data.

### Adult Abundance

We used one-way ANOVA to analyze the effect of treatment (initial larval combinations) on the total number and body mass of emerging adults. The numbers of adult *C. longiareolata* and *O. caspius* emerging from each cup vary together with similar error. Hence, emerging adult abundances of the two species may be regarded as random factors. We used MANOVA to analyze the effect of treatment on the number of emerging adults of the two species. Following [48] and [49] we applied Major Axis model-II regression to analyze the relationship among emerging adult species combinations. We report permutation test results and confidence intervals of the slope and intercept.

#### Adult body size

In analyzing effects of larval abundance on body size, we first analyzed the effect of larval density on per-cup average body size. However, this approach results in considerable loss of important variation. To gain further insight into these relationships we then explored how individuals’ body size varies with larval abundances (see below).

All four body size variables (wing, femur, and tibia lengths, and body mass) were highly and positively correlated. We thus used Principal Components Analysis (PCA) to combine all four size variables into a single composite variable to generally describe adult body size (see results). We then used the first principal component as a proxy of body size. Variation in resource availability through, e.g., competition during the larval stages, may affect larval growth rate. Such effects may interact with larval development time (duration) to affect consequent variation in adult body size, e.g., by allowing more time for resource accumulation. We thus analyzed how body size varied with larval abundances of both species, and with larval development time. We averaged the individual PC-1 scores in a cup for each species and sex separately. We used analysis of covariance to analyze the data with PC-1 as the response variable, treatment and sex as categorical predictors, and larval development time as a covariate. To approximate larval development time for each cup, we used the average larval development time of each species and sex in the different experimental cups separately.

#### Larval development time

We used Cox proportional hazards model to analyze species-specific effects of larval abundance on larval developmental time. This method allows the evaluation of effects of different predictors on occurrence rate of, e.g., adult emergence, independent of the time varying background mortality rate [50]. Using the Cox proportional hazards model allowed us to estimate a coefficient (β) for each one of the predictor variables and test for its significance. The exponent coefficient (e^β^), estimates the expected change in the event occurrence rate per one unit change in the covariate. For instance, e^β^ = 0.5 for competitor abundance means that the addition of a single competitor larva will result in halving the adult emergence rate, averaged over the entire experiment duration. To avoid pseudo-replication and to account for possible correlation between individuals within each experimental cup, we used a robust jackknife variance estimator grouped by observations (larvae) per cup [51]. In our analyses, larval development time (from hatching to adult emergence) was the response variable. This analysis was performed on the total number of larvae finally emerging as adults, so that sex and species could be determined accurately. We used sex (coded: male = 0, female = 1), initial intra- and inter-specific larval abundances, and the respective two-way interaction terms as predictor variables.

## Results

### Larval Abundance

#### Total number and body mass of emerging adults

The total number of emerging adults (considering the two species together) per cup (average = 20.6; 0.9 SE) and adult body mass (average = 13.055 mg; 0.559 SE) did not vary significantly with treatment (One-way ANOVA; F_4,25_ = 2.33, p = 0.08 and F_4,25_ = 0.31, p = 0.86 for total adult number and body mass, respectively).

#### Emerging-adult species combinations

**Figure 1:**
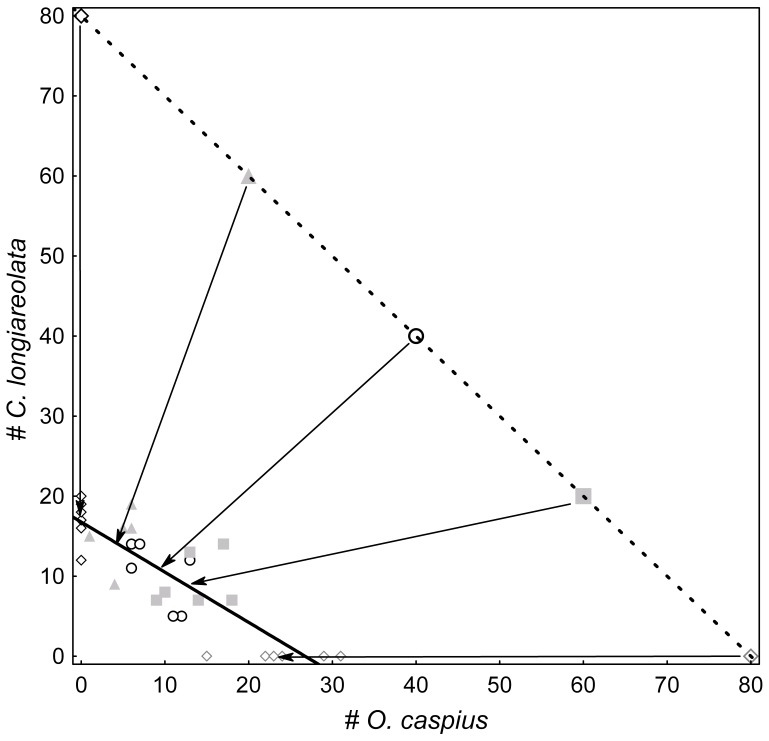
Initial vs. final species abundances. Initial (1^st^ instar) and final (emerging adults) abundance combinations of *O. caspius vs*. *C. longiareolata* in the experimental cups. Initial densities are connected by the dashed line (slope [−1]). The solid line represents the linear regression between the numbers of adults of the two species emerging from the experimental cups (slope −0.71). Arrows connect initial larval abundance combinations in the different treatments with the approximated centroid of the final species combinations of adults emerging from the respective experimental cups.

MANOVA indicated a significant effect of initial larval combinations (treatment) on the final adult abundance of both species (Wilks' lambda = 0.29612, F_4, 28_ = 5.8637, p = 0.0015; this analysis included only treatments in which the initial abundance of both species was greater than zero). Most differences among treatments occurred between treatments that differed the most in their initial larval combinations (Fig. 1). Final abundance combinations of adults of the two species emerging from each cup, were significantly and negatively related (Fig. 1; Major Axis model-II regression: p<0.0001 based on 9999 permutations): *#C. longiareolata = *17.6–0.71× *#O. caspius*. The slope of this model-II regression differed significantly from [−1] – its value at the onset of the experiment (95% C.I. of slope = [−0.90, −0.54]).

To examine possible sex-specific responses of larval survival to inter-specific abundances, we examined whether the sex ratio (the proportion of females out of the total emerging adults) of each species varied with the overall abundance of the emerging adults of the other species. In *O. caspius*, sex ratio did not significantly differ from 0.5 (average = 0.55; 0.04 SE, t_23_ = 1.23, p = 0.23), and was not related to the abundance of emerging *C. longiareolata* (linear regression; F_1,22_ = 1.17, p = 0.29). In *C. longiareolata*, sex ratio was significantly female biased, (average = 0.63; 0.05 SE, t_23_ = 2.94, p = 0.007), but was not related to the abundance of emerging *O. caspius* (linear regression; F_1,22_ = 0.07, p = 0.8).

### Larval Development Time

#### *Ochlerotatus caspius*

**Figure 2:**
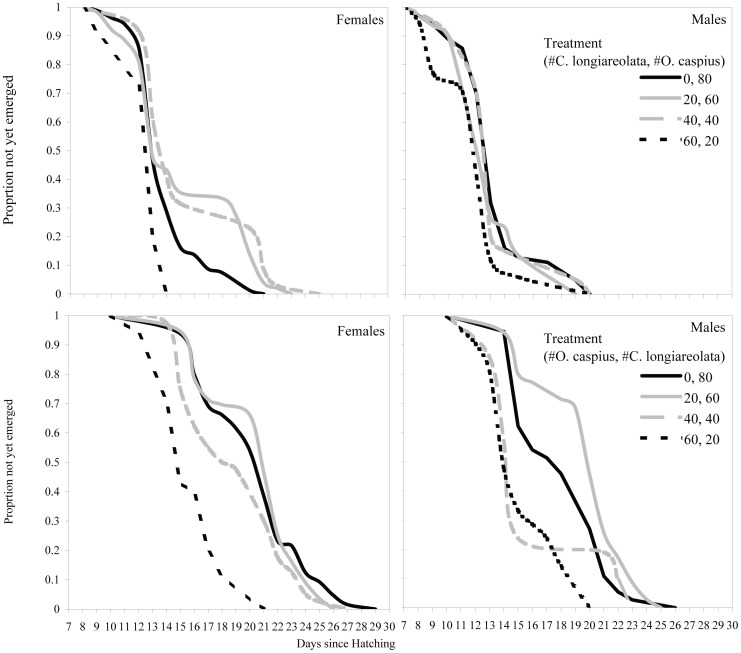
Sex-specific effects of treatment (initial larval combinations) on larval development time. A Kaplan-Meier fit for the relationship between the proportions of larvae not yet emerged, out of the final emerging population, and time since hatching. *O. caspius*: Upper panels, *C. longiareolata*: lower panels. Note, the legends are ordered to reflect increasing competitor abundance. Hence, exact line shade and pattern differ between the figures for the two species.

Males generally emerged before females (Table 2). Female larval development time varied with treatment, while male larval development time was indifferent to treatment (Fig. 2., Table 2).

**Table 2:** The effect of sex and initial larval abundances of larval development time.

Species	Variable	RegressionCoefficient (β)	P
***Ochlerotatus caspius***	Sex^a^	0.355	<0.0001
	Treatment	−0.009	0.3
	Sex ×Treatment	0.014	0.03
***Culiseta longiareolata***	Sex^a^	0.518	<0.0001
	Treatment	0.02	0.003

a *Female = 0, Male = 1*

Results of Cox proportional hazards model for the effect of sex and treatment (initial larval combinations) on the probability of adult emergence on time T (larval development time = time to emergence). For the analysis, initial larval combinations are ranked according to the relative competitor abundance (inter-specific larval abundance) for the focal species.

Interestingly, both sexes emerged in two distinct waves. Males started to emerge between days 8–9 in all treatments, peaking between days 10–14, during which time 80–90% of all emerging males had emerged. In all treatments, the second, smaller, emergence wave followed a 1–4 days delay in emergence, with the last males emerging between days 19–20.

Females *O. caspius* started to emerge between days 9–12, peaking between days 12–15, with no apparent treatment effect. However, the proportion of females emerging by that time varied with treatment (Fig. 2) and ranged between 65–100%. The second, smaller, emergence wave followed a treatment-dependent delay, with the last females emerging between days 14–25, also depending on treatment. Unlike in males, larval development time increased with increasing initial larval *C. longiareolata* abundance from 0 to 40, while a further increase, to 60, resulted in a decrease in larval development time.

Analysis using Cox Proportional Hazards Model indicates a significant effect of both sex and treatment on larval development time (Cox proportional hazard model, Wald test = 52.4, df = 3, P<<0.001; Robust Score test = 30.7, df = 3, P<0.002). While treatment itself was not significant, both sex and treatment×sex were significant, indicating sex-specific differences in the response of *O. caspius* larval development time to the initial larval combinations (Table 2).

#### *Culiseta longiareolata*

*C. longiareolata* (Fig. 2, Table 2) tended to emerge later than *O. caspius*. Like *O. caspius*, male *C. longiareolata* generally emerged before females. However, in *C. longiareolata*, larval development time of both sexes was affected by treatment. Male *C. longiareolata* started to emerge between days 11–14, depending on treatment, and generally kept a constant rate of emergence, with the last males emerging between days 20–26, again depending on treatment. Female *C. longiareolata* started to emerge between days 11–15, depending on treatment and generally kept a constant rate of emergence, with the last females emerging between days 21–29, again depending on treatment. In both sexes, larval development time generally decreased with increasing relative *O. caspius* larval abundance, especially in the treatments with relatively higher *O. caspius* abundance (40 and 60 larvae out of 80).

Analysis using Cox Proportional Hazards Model suggests significant effects of both sex and treatment on larval development time. The interaction term (treatment×sex) was not significant (P = 0.6), and was thus omitted from the analysis (Cox proportional hazard model, Wald test = 28.8, df = 2, P<0.0001. Robust Score test = 13.5, df = 2, P<0.002; Table 2).

### Adult Size

Using PCA, the first principal component (PC-1; Table 3) explained 94.5% and 94.3% of the size variation among *O. caspius* and *C. longiareolata* individuals, respectively, and was strongly and positively correlated with all four size variables. We thus used PC-1 as a proxy for body size in all further analyses.

**Table 3:** Adult size variables summarized by Principal Components Analysis (PCA).

Species	Eigenvalue	Total%Variance	Factor Loadings			
			Bodymass	Femur	Tibia	Wing
***O. caspius***	0.049	94.5	0.213	0.04	0.038	0.033
***C. longiareolata***	0.041	94.3	0.186	0.046	0.045	0.048

The first principal component (PC-1) of Principal Components Analysis (PCA) of the four body size variables (wing, femur, and tibia lengths and body mass) transformed to their Log_10_ values. The table details the eigenvalues of the covariance matrix, percent variance explained by the vectors and the factor coordinates of the variables, based on correlations (factor loadings).

#### Effect of treatment on average adult size

Controlling for treatment and larval development time, males of both species were significantly smaller than females. In both species, average adult size per-cup did not vary with treatment. However, while average adult size of *C. longiareolata* was not related to larval development time, the average *O. caspius* adult was larger with increasing larval development time (Table 4).

**Table 4:** The response of adult size to sex, treatment, and larval development time.

Species	Variable		df	MS	F	P
*O. * ***caspius***	Intercept		1	0.057	5.39	0.025
	Sex		1	0.3043	28.81	<0.0001
	Treatment		3	0.0005	0.05	0.99
	Larval development time		1	0.0572	5.41	0.025
	Error		40	0.0106		
***C. longiareolata***	Intercept		1	0.0004	0.04	0.85
	Sex	1		0.6604	61.5	<0.0001
	Treatment	3		0.0007	0.07	0.98
	Larval development time	1		0.0001	0.01	0.93
	Error	40		0.0107		

ANCOVA of the effect of sex, treatment (initial larval combinations), and larval development time on average per-cup body size as indicated by PC-1: **a)** *O. caspius* (whole model: F_5,40_ = 8.69, R^2^ = 0.52, p<0.0001) **b)** *C. longiareolata* (whole model: F_5,40_ = 13.53, R^2^ = 0.63, p<0.0001).

### Individual Variations in Body Size (Relationship between Treatment, Larval Development Time, and Individual Adult Size)

#### *Ochlerotatus caspius*

**Figure 3:**
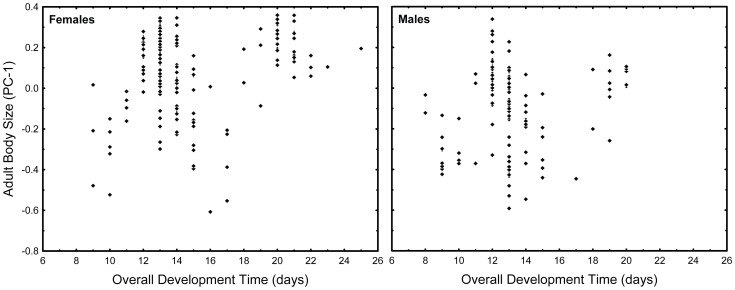
Adult size variation in *O. caspius*. Variation in adult size (PC-1) *O. caspius* with respect to larval development time (time from hatching to adult emergence). Data pooled over all experimental cups and treatments.

Pooled over all experimental cups and treatments, adult body size suggests a bimodal pattern with respect to larval development time, especially in females (Fig. 3). This pattern clearly reflects the two emergence waves described above for larval development time (Fig. 2).

To analyze the relationship between adult size, larval development time and treatment, we divided the time axis into two phases, corresponding with the two emergence waves analyzed above. Since the timing of the first wave did not vary with treatment (Fig. 2), we set the first time phase from the first day of emergence through day 14, and the second time phase from day 15 through day 25– the last day of emergence (see Fig. 2 and relevant text above).

**Figure 4:**
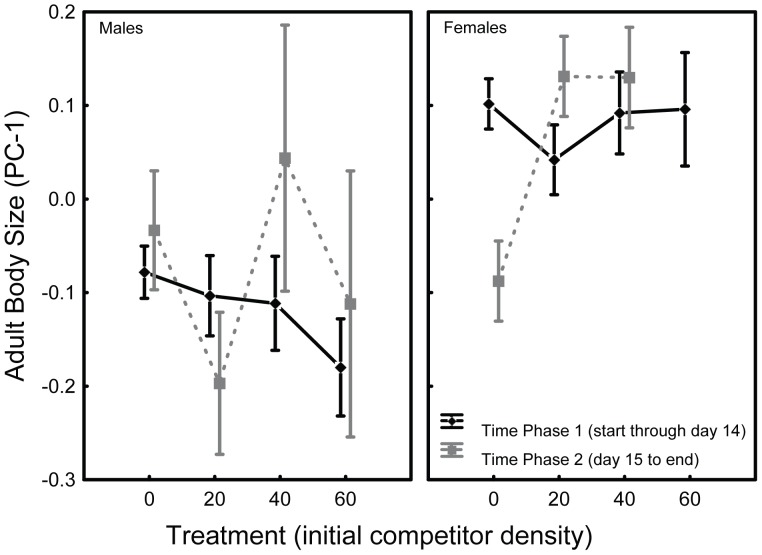
Sex-specific variation in adult size in the two emergence waves. *O. caspius* variation in body size with respect to time phase and treatment (initial larval combinations, ordered according competitor density). The two phases correspond with the two emergence waves described above (Fig. 2). Error bars denote ±1S.E.

Time phase had no significant effect on female body size. However, the treatment effect was marginally significant and the treatment×time phase interaction was highly significant (Table 5, Fig. 4). Figure 4 suggests that the size of female *O. caspius* emerging during the 1^st^ time phase was not affected by treatment. While so, *O. caspius* females emerging during the 2^nd^ time phase were smaller in the absence of inter-specific competition, but similar in size to females emerging on the 1^st^ wave in the presence of *C. longiareolata*. With respect to size, *O. caspius* males were indifferent to treatment, time phase and their interaction (Table 5 and Fig. 4).

**Table 5:** ANOVA of the effect of treatment and time phase on *O. caspius* size.

Sex	Variable	df	MS		F	P
***Females***	Intercept	1	0.6007		15.33	0.0001
	Treatment	3	0.1027		2.62	0.053
	Time Phase	1	0.0148		0.38	0.54
	Treatment × Time Phase	2	0.3058		7.8	0.0006
	Error	168	0.0392			
***Males***	Intercept	1	0.4145		9.84	0.0021
	Treatment	3	0.0535		1.27	0.29
	Time Phase	1	0.0211	0.5		0.48
	Treatment × Time Phase	3	0.0366	0.87		0.46
	Error	118	0.0421			

#### *Culiseta longiareolata*

In *C. longiareolata*, we found no consistent division of the larval development time-line among the different treatments (see Fig. 2 and related analyses above). We thus used ANCOVA to analyze variation in adult size of individual *C. longiareolata* with respect to treatment and larval development time. Our results (Table 6) suggest that longer larval development time corresponds to larger females and that this effect is stronger (steeper) in treatments with higher competitor (*O. caspius*) abundance (time × treatment interaction: p = 0.0094). Male *C. longiareolata* were also larger with increasing larval development time, but we found no effect of treatment.

**Table 6:** *C. longiareolata*, ANCOVA of the effect of treatment and time phase (adult emergence) on size.

Sex	Variable	df	MS	F	P
***Females***	Intercept	1	0.183	6.81	0.01
	Treatment	3	0.1191	4.43	0.0049
	Overall time	1	0.2828	10.52	0.0014
	Treatment × Overall time	3	0.1058	3.94	0.0094
	Error	186	0.0269		
***Males***	Intercept	1	0.4854	17.44	<0.0001
	Treatment	3	0.0539	1.94	0.13
	Overall time	1	0.1934	6.95	0.01
	Error	106	0.0278		

## Discussion

Our data indicate sex-specific effects of intra- and inter-specific larval abundances on larval-mosquito development strategy and survival. In the context of this experiment, *C. longiareolata* was the superior competitor suggesting its importance in the temporary pond ecosystem (see also [40]–[42]). The total number of emerging adult mosquitoes (the two species combined) was substantially lower than the initial larval numbers, but was unrelated to treatment. This is consistent with compensatory density dependence [3], [52], and indicative of constant carrying capacity among experimental units and strong resource competition. Indeed, adult emergence of the two species was negatively correlated with a slope significantly different from unity, indicating asymmetric larval competition in favor of *C. longiareolata*. *C. longiareolata* may also act as an intra-guild predator ([40], [53], [54]), and there is evidence that its larvae potentially prey on *O. caspius* (and other mosquito larvae)([53]–[55]). However, this usually occurs when there are substantial size differences in favor of *C. longiareolata* larvae ([40], [53], [55]). In the present experiment, the larvae of the two species were of similar stages, and we did not find any evidence of predation. The fact that there is no difference between treatments in the overall number of emerging adults also suggests that *C. longiareolata* larvae do not prey upon *O. caspius* larvae, unless predation and competitive effects are completely compensatory.

Nonetheless, *C. longiareolata* larvae are scavengers, and often feed on dead larvae of either species. If their competitive effect results in decreased survival of *O. caspius*, then they will have more carcasses to feed on, and hence, indirectly benefit from the interaction with *O. caspius*. This can substantially reduce the negative competitive effect of *O. caspius* on *C. longiareolata*, resulting in a highly asymmetric competitive effect, or may even result in net antagonistic effect (+/−) rather than the classic interspecific competitive effect (−/−). Sex-specific density-dependent survival in mosquitoes have been reported to be species- and context-dependent, ranging from equal for both sexes [16], [19], stronger for males [56], or for females [44]. In this study, adult females of both species were generally more abundant than males, with no sex-specific differences in the response to inter-specific competition.

Larval mosquitoes also modified their development strategy in response to variations in inter- and intra-specific larval abundances (treatment). Furthermore, this response was sex-specific, consistent with other studies [9], [16], [19], [44], [56]–[58]. In both species of this study, male and female emergence overlapped considerably, with males generally preceding females by 1–2 days. Males of both species were also generally smaller than females. Larval development time is expected to be sex-specific, context-dependent, and at least partially determined by trade-offs between reproductive benefits gained by continued larval growth (through nutrient accumulation), and costs such as deterioration of habitat conditions (e.g., waste and competition) and risk of death (e.g., desiccation, pathogens, and predators) [8], [44], [59], [60]. To advance in instar stage and finally complete metamorphosis, larvae must first exceed certain nutrient-dependent developmental thresholds [11], [44], [61]. As females’ reproductive success improves more than males’ by being larger, their minimal and optimal larval developmental time is expected to be longer and likely more sensitive to deterioration of growth conditions [9], [44], [59], [62], [63]. Indeed, our analyses indicate such complex, species-specific and sex-specific relationships between larval densities, larval development time, and adult size.

In *O. caspius,* both sexes emerged in two distinct waves - a large wave, followed by a second, much smaller one. The timing of the first wave and the size of the resulting adults were unrelated to treatment. In males, the magnitude of the first wave did not vary with treatment. However, in females the magnitude of the first wave decreased with increased inter-specific abundance from zero to 40 larvae. While so, in the highest inter-specific abundance (60 *C. longiareolata* larvae), all adult females emerged in a single wave, timed with the first wave of the other treatments. In males, the timing, magnitude, and emerging adult size of the second wave were also unrelated to treatment. In females, however, the onset of the second wave and the resulting adult size varied with treatment. The time delay preceding the second wave of emerging females ranged from zero, with no competitor larvae present, to 4 days in the presence of 40 competitor larvae. Interestingly, the average body size of second-wave females was smaller in the absence of *C. longiareolata*, but was similar in size to first wave females in its presence. With 60 inter-specific competitor larvae, all emerging adult females completed their metamorphosis by the end of the first wave. However, observations indicate that *O. caspius* larvae were still present in the experimental cups beyond this time, though none of these emerged. This larval development pattern may simply reflect small developmental advantages, such as slightly different hatching times. Individuals that hatch early may have competitive advantage over individuals that hatch slightly later, resulting in developmental advantages. If so, the two waves may represent different responses to environmental context, in which early-hatching individuals develop fast, while late-hatching individuals develop slowly until the first wave clears out. However, this mechanism would require two discrete (or nearly so), very shortly spaced, hatching events.

[64] and [9] suggest that larvae of container-breeding mosquito species, which can be subject to long periods of food shortage, are able to substantially extend their development period relative to ground-pool mosquitoes, but have a substantially lower maximal development rate than the latter. The work of Bradshaw et al. [60] indicate that mosquito development rate is at least partially heritable, with substantial geographic variation with respect to environmental conditions. Possibly, there are two distinct strategies of larval development rate in our *O. caspius* population, each with a certain range of plasticity. Larvae with basically high development rates comprise the first emergence wave, while larvae with lower basal development rates comprise the second emergence wave. As discussed in [44], and consistent with our *O. caspius* males, they may have exceeded the developmental optima before competition arising specifically from *C. longiareolata* deteriorated their growth conditions, and hence were unaffected by *C. longiareolata* presence with respect to larval developmental time. Similarly, females with fast development rate were also unaffected by *C. longiareolata* presence. While so, females with low basal development rates were still in the water when competition arising specifically from *C. longiareolata* became influential on larval development. Hence, the larval development time of these females was increasingly retarded in response to increasing densities of *C. longiareolata*. This may have occurred because it took the larvae more time to achieve minimal developmental thresholds, or because their optimal developmental time had been shifted, or both. Interestingly, beyond a certain time delay, these females were probably unable to complete metamorphosis (see [11], [61]). Hence, all adult females emerging from cups in the treatment with the highest competitor densities are those that emerged in the first wave, while the second wave females, which possibly delayed their emergence beyond this limit, died as larvae. Consequently, larval development time was shorter in this treatment, not necessarily because larval development was generally faster, but possibly because individuals that did not manage to emerge on the first wave did not emerge at all. Based on the size distributions of the emerging females and the otherwise consistently negative effect of *C. longiareolata* on *O. caspius*, we suggest that the size increase in the presence of *C. longiareolata* results from selection against smaller-sized females in the second wave, rather than through improved growing conditions (see e.g., [45]). Possibly, in the absence of *C. longiareolata*, “lower quality” females that cannot survive extended larval development time pupate as soon as they achieve minimal pupation threshold and are consequently smaller adults. Under the strong competitive conditions exerted by the presence of advanced-stage *C. longiareolata* larvae, prior to the second emergence wave, these “low-quality” individuals could not achieve minimal pupation threshold and did not emerge at all. Alternatively, if state-dependent predation by *C. longiareolata* occurs, these lower quality females would be the most vulnerable. Females of the “slow” strategy could withstand extended developmental periods, acquired necessary nutrients for optimal (larger) size even under intense competitive environment, at the price of extended larval development time.

Unlike *O. caspius*, *C. longiareolata* larval development time was generally constant, with no consistent within-treatment change in pattern. Nonetheless, emergence rate was significantly affected by treatment, with larval development time negatively correlated with *O. caspius* initial densities. This pattern may probably indicate the release from intense intra-specific competition, rather than facilitation by *O. caspius,* since higher *O. caspius* initial densities are directly associated with lower *C. longiareolata* initial densities. This is also in accordance with our findings concerning the numeric relationship between *C. longiareolata* and *O. caspius* adults emerging from our experimental cups. Nonetheless, as discussed above, *C. longiareolata* larvae are scavengers and potential intra-guild predators. If their competitive effect results in decreased state and survival of *O. caspius*, then they may experience increased feeding opportunity along with decreased intra-specific competition, and hence shorter larval development time, higher survival and larger body size at emergence.

The average, per cup, size of adult *C. longiareolata* was neither related to larval densities nor to average larval development time. However, our analysis of individual variation in body size suggests otherwise. Consistent with previous findings [8], [9], [65]–[67], our analysis indicates that males' and females' body size were positively correlated with their larval development time. Furthermore, while males were not affected by treatment in this respect, female body size increased more steeply with larval development time in the presence of higher *O. caspius* initial abundances (corresponding to higher inter- but lower intra-specific initial abundances). Possibly, female *C. longiareolata* benefit more from extending their larval development time under lower intra-specific competition [9], [44], [62], [63].

*C. longiareolata* and *O. caspius* co-occurrence is probably maintained by both spatial and temporal mechanisms. First, habitat segregation at the landscape scale occurs with *C. longiareolata* dominating freshwater temporary habitats, at their early successional stages, and *O. caspius* inhabiting, in addition to freshwater habitats, brackish and saline ones [26]. Additional mechanisms work within these habitats to maintain coexistence with other competitors (mosquito species) [68]–[70]. In water bodies in which the two species co-occur, *O. caspius* probably finds partial refuge in faster developmental rate. Some individuals possibly find refuge in extending larval development time, hence maintaining final size in the price of slower development rate and the accompanying hazards of prolonged aquatic natal stages. In the presence of *C. longiareolata*, the two development strategies result in overall similar sizes. If these differences are genetically based, intermediate competition from *C. longiareolata* may impose disruptive selection pressure with respect to larval developmental time, while exerting stabilizing selection on size (selecting against the small ones of the second wave). While so, intense competition from *C. longiareolata* may strongly select against the slow development rate, favoring significantly shorter generation time.

More importantly, these two developmental strategies may play roles in *O. caspius* population dynamics and intra-specific competition. *O. caspius* hatching, following even single habitat inundation, is asynchronous in that not all eggs hatch together [71]. This creates distinct co-occurring stage differences with the younger stages competitively inferior. Individuals of younger stages may find temporal refuge in prolonged development, slowly accumulating enough resources for optimal size, but more importantly, by delaying development until the older, superior competitors pupate and competitive pressure is relieved. The full details of *O. caspius* development strategies are yet unknown. However, they may possibly also relate to its unusual egg laying strategy. For example, *O. caspius* eggs are laid on water or in wet soil above the water surface ([9], [35], Tsurim and Silberbush, personal observations). Those in soil enter diapause. From those that are laid on water, some drift to the edge, desiccate and enter diapause, and the rest hatch within a few days. Larvae resulting from the latter eggs hatch into water bodies in more advanced successional stages, often with unfavorable competitive conditions imposed by e.g., larvae of more advanced developmental stages. Being able to develop slowly may allow sufficient resource accumulation for optimal size or provide temporal refuge until older larvae pupate and growing conditions improve (competitive release).
